# Mechanism of MyD88S mediated signal termination

**DOI:** 10.1186/s12964-021-00811-1

**Published:** 2022-01-20

**Authors:** Katarzyna Pustelny, Katarzyna Kuska, Andrzej Gorecki, Bogdan Musielak, Ewelina Dobosz, Benedykt Wladyka, Joanna Koziel, Anna Czarna, Tad Holak, Grzegorz Dubin

**Affiliations:** 1 Malopolska Centre of Biotechnology, Jagiellonian University, 30-387 Kraków, Poland; 2 Faculty of Biochemistry, Biophysics and Biotechnology, Jagiellonian University, 30-387 Kraków, Poland; 3 Faculty of Chemistry, Jagiellonian University, 30-387 Kraków, Poland

**Keywords:** MyD88, MyD88S, Toll-like receptor (TIR), Interleukin-1 receptor (IL-1R), Signaling, Innate immunity

## Abstract

**Background:**

A universal adaptor protein, MyD88, orchestrates the innate immune response by propagating signals from toll-like receptors (TLRs) and interleukin-1 receptor (IL-1R). Receptor activation seeds MyD88 dependent formation of a signal amplifying supramolecular organizing center (SMOC)—the myddosome. Alternatively spliced variant MyD88S, lacking the intermediate domain (ID), exhibits a dominant negative effect silencing the immune response, but the mechanistic understanding is limited.

**Methods:**

Luciferase reporter assay was used to evaluate functionality of MyD88 variants and mutants. The dimerization potential of MyD88 variants and myddosome nucleation process were monitored by co-immunoprecipitation and confocal microscopy. The ID secondary structure was characterized in silico employing I-TASSER server and in vitro using nuclear magnetic resonance (NMR) and circular dichroism (CD).

**Results:**

We show that MyD88S is recruited to the nucleating SMOC and inhibits its maturation by interfering with incorporation of additional components. Biophysical analysis suggests that important functional role of ID is not supported by a well-defined secondary structure. Mutagenesis identifies Tyr116 as the only essential residue within ID required for myddosome nucleation and signal propagation (NF-κB activation).

**Conclusions:**

Our results argue that the largely unstructured ID of MyD88 is not only a linker separating toll-interleukin-1 receptor (TIR) homology domain and death domain (DD), but contributes intermolecular interactions pivotal in MyD88-dependent signaling. The dominant negative effect of MyD88S relies on quenching the myddosome nucleation and associated signal transduction.

**Video abstract**

**Supplementary Information:**

The online version contains supplementary material available at 10.1186/s12964-021-00811-1.

## Background

Innate immune system provides the forefront host defense against invading pathogens. Pattern recognition receptors (PRRs) recognize molecules characteristic for pathogens enabling rapid response to emerging threats [1, 2]. Toll-like receptors (TLRs), TLR1-10, play an eminent role among PRRs [3]. TLRs send warning signals in response to a diverse range of pathogen specific molecules including bacterial lipopeptide (TLR2 associated with TLR1 or TLR6), lipopolysaccharide (TLR4), bacterial flagellin (TLR5), viral dsRNA (TLR3), viral or bacterial ssRNA (TLR7 and TLR8) and CpG-rich unmethylated DNA (TLR9) [4]. Despite functional diversity, TLRs share common structural organization and all involve intracellular Toll-interleukin-1 receptor (TIR) domains for signal transduction [5]. TIR domains are even more universal. They are additionally involved in signal transduction by interleukin-1 family receptors and constitute functional domains of a number of cytosolic adaptor proteins, which link receptors and intracellular events. Five TIR-domain-containing adaptor proteins are involved in inflammatory signal transduction: myeloid differentiation primary response 88 (MyD88), MyD88-adaptor-like (MAL; known as TIRAP), TIR-domain-containing adaptor protein inducing IFNβ (TRIF; known as TICAM1), TRIF-related adaptor molecule (TRAM; known as TICAM2) and sterile-alfa and armadillo motif-containing protein (SARM) [6].

MyD88 is the central adaptor of innate immunity, integrating signaling via IL-1 receptor (IL-1R) and all TLRs, except TLR3. MyD88L (long) contains three domains: the N-terminal death domain (DD), the central linker region known as the intermediate domain (ID) and the C-terminal TIR domain [7]. MyD88-dependent signaling relies on an assembly of a supramolecular organizing center (SMOC), a unique multiprotein complex, known as the myddosome [8, 9]. The SMOC is formed in response to pathogen mediated stimulation of TLRs/ligand mediated stimulation of IL-1 receptor [10]. Intracellular TIR domains of stimulated receptors transiently interact with TIR domains of MyD88 providing a nucleation point for DD-mediated assembly of the myddosome. Homotypic interactions among MyD88 death domains constitute the core of the myddosome which recruits DD-containing IRAK kinases (IRAK4, IRAK1/2) by virtue of heterotypic DD interactions. Myddosome formation initiates the phosphorylation cascade which propagates the signal into the nucleus via activation of NF-κB and activator protein 1 (AP-1) ultimately leading to the production of inflammatory mediators.

Timely immune response is essential to combat the invading pathogens, but excessive or chronic inflammation may have devastating consequences to the host. Efficient, multilevel control is embedded into the innate responses and includes, among others, switching of splicing variants with opposing functions [11]. Human *MyD88* gene maps to chromosome 3p21.3-p22 and contains five exons [12, 13]. The first exon encodes a complete DD (amino acids 1–109; GenBank no. U70451), the second exon encodes the ID (110–155) and the last three exons encompass the TIR domain (156–296). Two major splice variants of *MyD88* have been identified: full length protein MyD88L and a shorter variant, MyD88S (short), missing exon 2 which results in an in-frame deletion of entire ID. MyD88L is the primary functional product at short stimulation times and transduces the activating signals. MyD88S is detected only at continuous stimulation with bacterial products or proinflammatory cytokines [14, 15] and acts as a dominant negative regulator of IL-1β and LPS-induced NF-κB activation, silencing the prolonged immune response. Reportedly, the antagonistic activity of MyD88 splicing variants is related to their differential ability to recruit IRAK4 [16]. Failure to recruit IRAK4 to the myddosome precludes IRAK1/IRAK2 phosphorylation terminating the signal.

Recognition of the opposing functional roles of MyD88 splicing variants is not supported by mechanistic understanding. In contrast to relatively well-established role of DD and TIR domains in MyD88 activity, the involvement of ID is poorly understood at the structural level. Crystal structure of a myddosome fragment (complex of DDs of MyD88, IRAK4 and IRAK2; PDB ID: 3MOP) [9] and cryo-EM structure of helical filament of MyD88 DDs (PDB ID: 6I3N) [17] have only defined the arrangement of the initial part of ID. Here, using domain fragmentation and mutational analysis coupled to functional characterization in myddosome formation and signal transduction assays, and biophysical approaches, we define the mechanistic role of ID in myddosome assembly. Assessing the intermolecular interactions guiding the nucleation of the myddosome, we provide the mechanistic rationale for the dominant negative effect of MyD88S.

## Results

### Intermediate domain (ID) has essential function in MyD88-dependent signaling

**Fig. 1:**
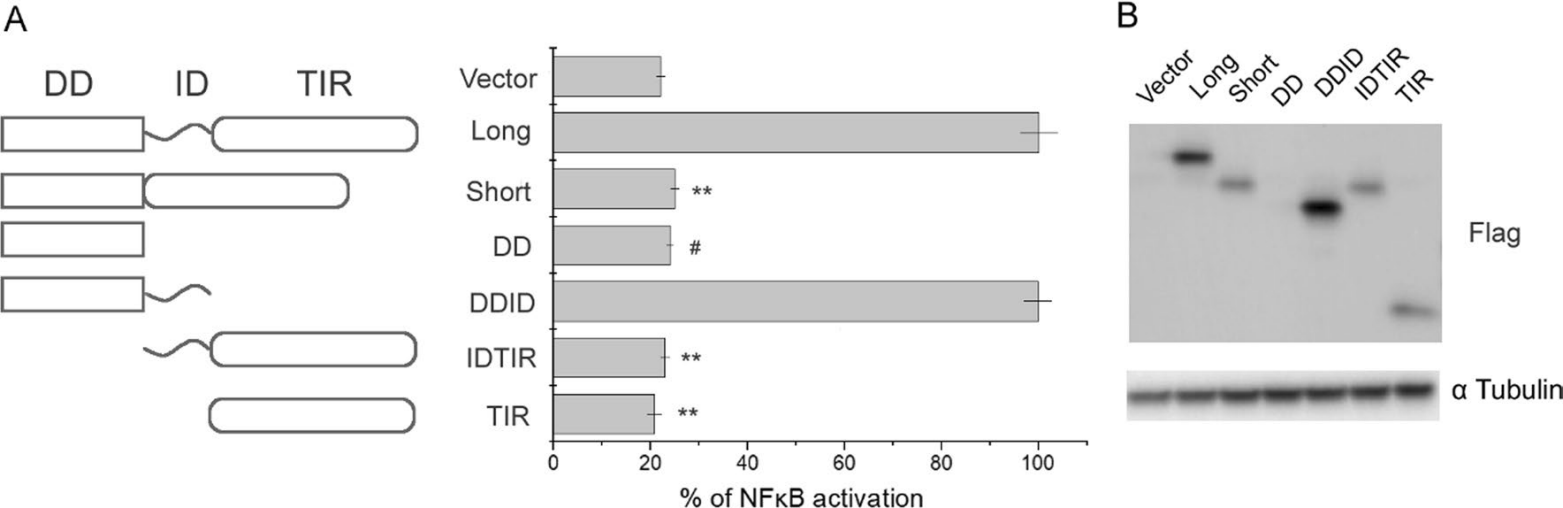
Intermediate domain (ID) of MyD88 is essential for downstream signaling. **A** Effect of overexpression of different domains of MyD88 and their combinations (depicted schematically to the left) in HEK293-I3A (MyD88^−/−^) cells on NF-κB-driven reporter expression. The response was normalized to MyD88L signal. Values are presented as means ± SD from triplicates. Statistical significance was determined by Student’s t-test (**p < 0.001). # indicates no expression. **B** Semiquantitative analysis of transgene expression by Western Blot (α-Flag). α-Tubulin was used as the loading control

It has been demonstrated earlier, that myddosome signaling converges at NF-κB activation and that MyD88L overexpression in a cell results in spontaneous myddosome assembly and signaling even in the absence of receptor stimulation [18]. To assess the functional characteristics of MyD88 domains we used a well validated overexpression-coupled NF-κB reporter assay [19–23]. Tested domains and their combinations (Fig. 1A) were overexpressed in MyD88-defficient HEK293-I3A cell line [20] to exclude the potential influence of endogenous MyD88. Overexpression of MyD88L, containing DD, ID and TIR domains, resulted in stimulation of NF-κB driven reporter expression while overexpression of MyD88S, containing only DD and TIR domains, had no effect (Fig. 1A) despite reaching protein expression levels comparable to MyD88L (Fig. 1B). These results demonstrate that the ID is essential for spontaneous MyD88 signaling in the absence of receptor stimulation. Overexpression of TIR domain itself had no effect on NF-κB-driven reporter expression. Overexpression of the C-terminal part of MyD88 (IDTIR) containing ID and TIR domains had no effect on transgene expression, demonstrating that ID domain is not sufficient to induce spontaneous myddosome assembly. We were unsuccessful in overexpressing the DD itself in our system (Fig. 1B), but earlier study demonstrated that it is insufficient to induce NF-κB-responsive promoter [24]. Overexpression of N-terminal part of MyD88 (DDID) containing DD and ID resulted in stimulation of reporter expression comparable to that obtained at overexpression of MyD88L, demonstrating that ID appended DD is sufficient to nucleate kinetically driven myddosome assembly, while TIR domain is dispensable in this process (the assay does not account for upstream signaling where TIR domains are indeed essential) [25, 26].

### Only a small fragment of ID mediates its function in myddosome assembly

**Fig. 2:**
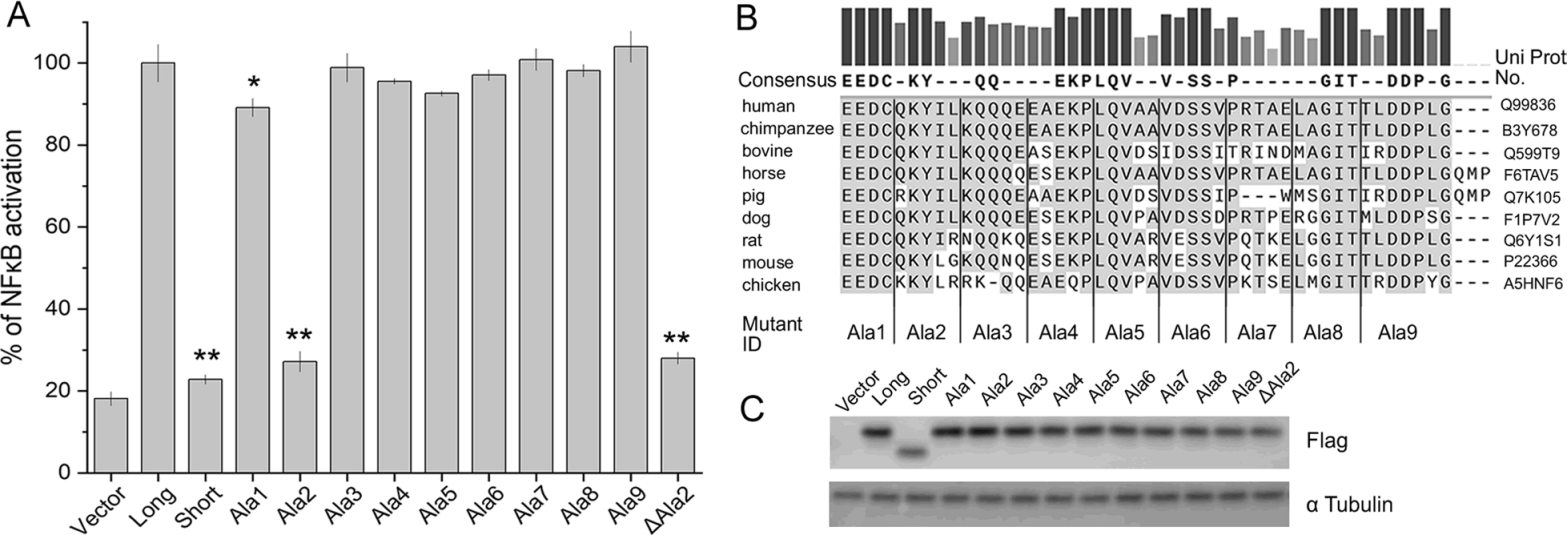
Residues Gln114-Leu118 mediate ID function in MyD88 dependent signaling. **A** Stretches of consecutive residues (indicated in panel **B**) in MyD88 ID were exchanged to alanine and the effect of mutant overexpression on NF-κB driven reporter expression was evaluated in HEK293-I3A cells. Response was normalized to MyD88L. Values are presented as means ± SD from triplicates. Statistical significance was determined by Student’s t-test (*p < 0.05, **p < 0.001). **B** Conservation of MyD88 ID across species and residues selected for alanine substitution. **C** Level of transgene expression in experiment shown in (**A**) determined by Western blot. α-tubulin was used as the loading control

Having demonstrated the essential role of the ID in myddosome assembly we wanted to gain mechanistic insight. To delineate the regions of functional significance, consecutive fragments of ID were substituted with alanine residue stretches of corresponding length (Fig. 2B) and overexpressed in the context of MyD88L while monitoring NF-κB-responsive reporter gene expression. All tested mutants were expressed at comparable level as demonstrated by Western Blot (Fig. 2C). Overexpression of all, but two, tested mutants induced the reporter expression at the level comparable to MyD88L (Fig. 2A) suggesting that the amino acid composition of the major part of ID is irrelevant for its function in sustaining downstream signaling. Only alanine substitution of residues Gln114-Leu118 (Ala2) significantly affected the reporter expression, compared to the wild type MyD88L—the mutant has lost its ability to activate the reporter. The activating potential of Glu110-Cys113 (Ala1) mutant was slightly reduced compared to the wild type, but the effect barely reached statistical significance. This demonstrates that despite the conserved nature of the intermediate domain of MyD88 (Fig. 2B), only residues Gln114-Leu118 have a significant role in myddosome formation. Deletion of Gln114-Leu118 (ΔAla2) had a similar effect on transgene expression as the alanine substitution—the deletion mutant lost its ability to activate the reporter, again signifying the role of Gln114-Leu118 in myddosome assembly.

The effect of alanine substitution of residues Gln114-Leu118 (Ala2) or deletion of said residues (ΔAla2) was comparable to the effect of MyD88S (where ID is completely absent) and in fact the empty vector control. These results suggest that residues Gln114-Leu118 provide the majority of interactions within the intermediate domain necessary for myddosome assembly and downstream signaling in tested conditions.

### ID is largely unstructured

Interspecies sequence conservancy of ID could suggest a conserved structure of this domain. To the contrary, our observation that the amino acid sequence of the major part of ID is irrelevant to its function suggested its unstructured nature. To address this apparent inconsistency we characterized the folding of ID in more detail.

In silico prediction of the structure of ID (as a 46 amino acid residues isolated peptide) of MyD88 with I-TASSER [27–29] suggested primarily flexible random coil organization. Only the initial 14 amino acid residues (corresponding to Glu111-Glu124 of MyD88L) were predicted to assume an α helical fold consistent with prior crystal and cryo-EM structures of MyD88 DD, were the several initial residues of ID were defined as a continuation of the C-terminal α helix of DD.

**Fig. 3:**
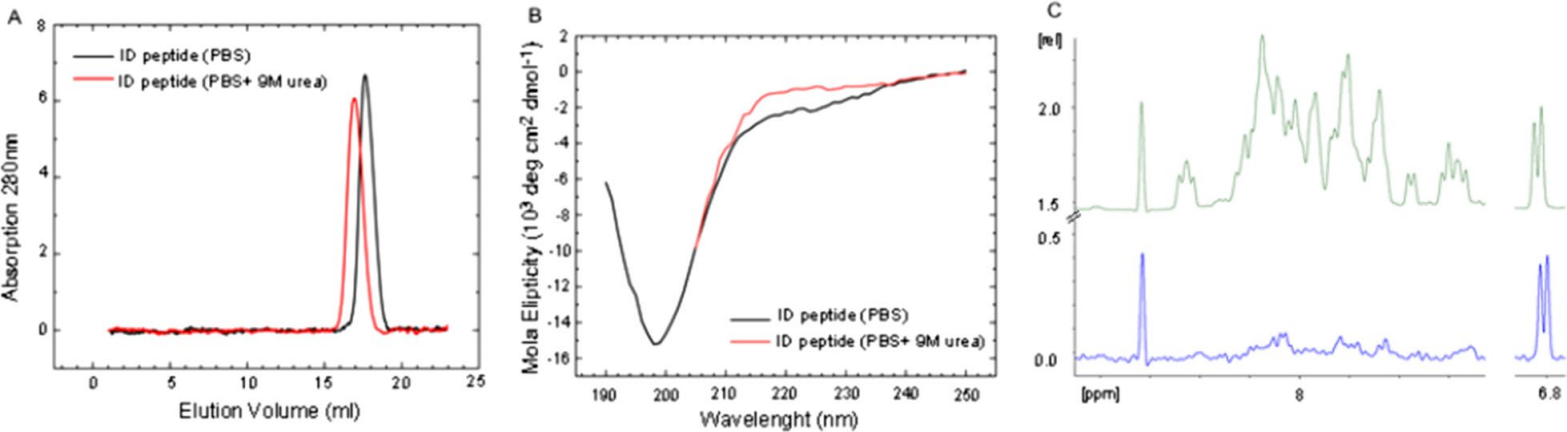
Structural characterization of ID. **A** Size exclusion profiles of 46 amino acid peptide corresponding to ID (Glu110-Gly155) of MyD88 suggest that the peptide is characterized by a secondary structure which is affected by a chaotropic agent. **B** Circular dichroism suggests a primarily intrinsically disordered nature of ID with low content of secondary structures which may by further unfolded in presence of a chaotropic agent. **C** Amide region of ^1^H NMR spectrum of ID in water (top) and 91% D_2_O (bottom). Deuterium exchange of amide protons is seen as hydrogen signal disappearance. A fragment of the aliphatic region is shown for reference (right)—aliphatic hydrogens do not exchange, signal remains unaffected

To evaluate in silico predictions we synthesized a peptide corresponding to the intermediate domain of MyD88 (Glu110-Gly155) and assessed its structure in solution. The hydrodynamic radius of the peptide determined by size exclusion chromatography was different in native and denaturing conditions. Partition coefficients (K_AV_; 0.697 and 0.649, respectively) and related Stokes radii (R_S_ 2.03 and 2.43 nm, respectively) indicated structure relaxation in denaturing conditions, suggesting that some internal interactions stabilize the structure of the peptide in native conditions (phosphate buffered saline) (Fig. 3A).

Circular dichroism (CD) spectrum of ID in PBS was typical of an intrinsically disordered peptide with a deep minimum at c.a. 200 nm. Nonetheless, the spectrum could have been deconvoluted into 60% share of disordered structure, but also 25% of beta strands, suggesting that ID is characterized by some secondary structure elements. Accordingly, a CD spectrum recorded at denaturing conditions showed higher ellipticity in the range of 210–230 nm indicating increased disorder (Fig. 3B).

^1^H NMR monitored hydrogen–deuterium exchange experiment demonstrated that the secondary structures within ID are not stable in over a dozen minutes timescale. 1D proton NMR spectrum of ID peptide in water showed a complicated signal from amide protons while the signal disappeared almost completely when the peptide was dissolved in 91% D_2_O signifying that all the amide hydrogens freely exchange for deuterium (Fig. 3C). This suggests that none of the amide protons within ID is involved in intermolecular hydrogen bonds stable in over a dozen minutes timescale.

Collectively, our results argue that the structure of isolated ID is primarily a random coil with residual and unstable secondary structure elements.

### Substitution of only five residues in the ID of MyD88L mimics the dominant negative effect of MyD88S

**Fig. 4:**
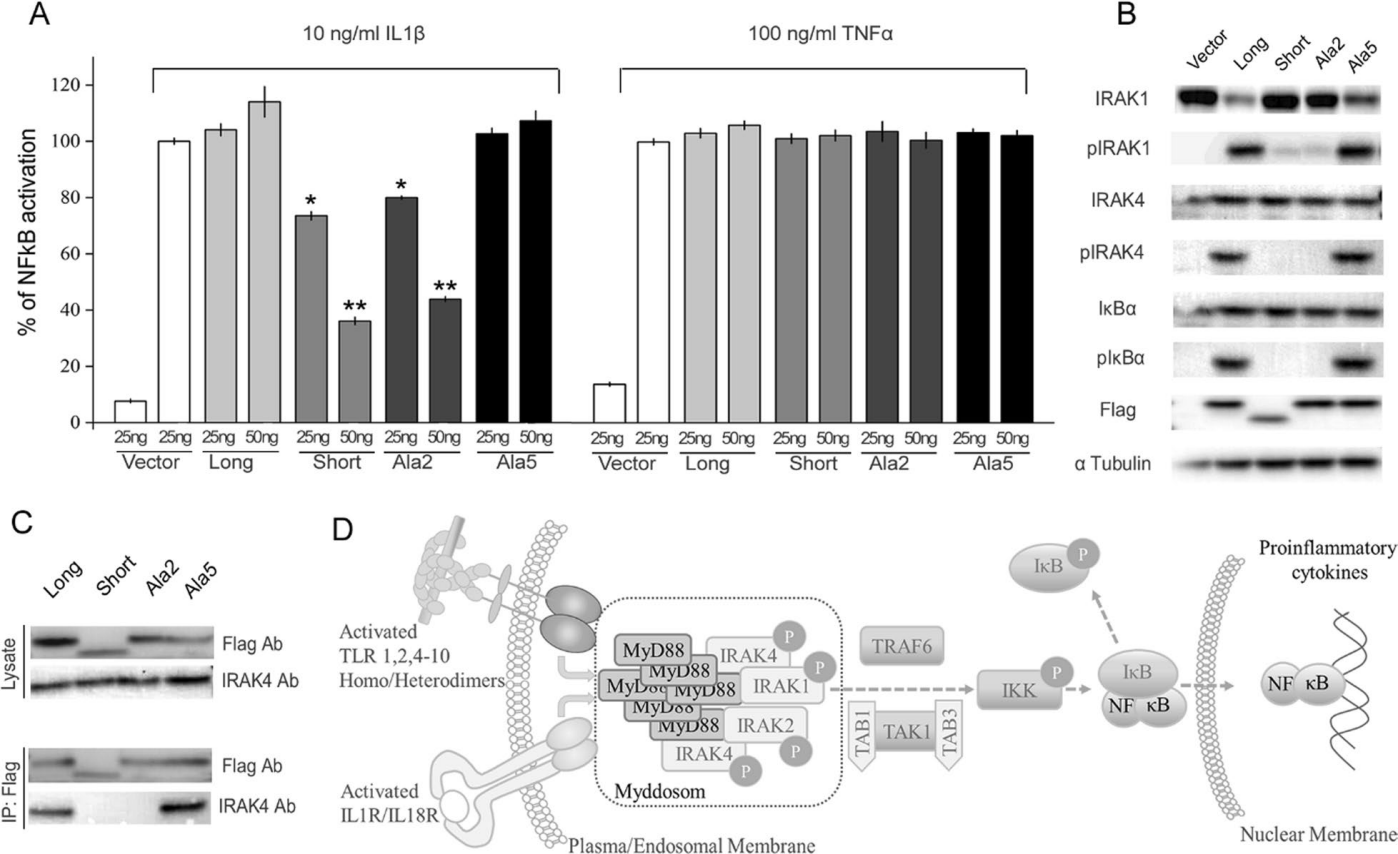
MyD88(114-118)Ala (Ala2) mimics the dominant negative effect of MyD88S. **A** Effect of overexpression of indicated MyD88 variants on IL-1β (10 ng/ml) and TNFα (100 ng/ml) induced stimulation of NF-κB responsive reporter expression tested in the presence of endogenous MyD88 in HEK293T cells. Data are normalized to the response of empty vector transfected and cytokine stimulated cells, and are shown as means ± SD of triplicate samples. Statistical significance was determined by Student’s t-test (*p < 0.05, **p < 0.001). **B** Effect of overexpression of indicated Flag-tagged MyD88 variants on the phosphorylation state of selected MyD88 effectors in HEK293-I3A cells. **C** Interaction of MyD88 variants (Flag-tagged) with endogenous IRAK4 was analyzed by co-immunoprecipitation on anti-Flag beads and evaluated using anti-Flag and anti-IRAK4 antibodies. **D** Diagram of simplified MyD88 signaling

The robust overexpression model of myddosome assembly is useful for evaluating the downstream effects, but poorly resembles the physiological triggers involved in myddosome formation. This is especially evident when TIR domain is considered, which is not required for signaling in the overexpression model, but was previously demonstrated indispensable in receptor mediated signaling [25, 26]. Therefore, we re-evaluated the effect of MyD88(114–118)Ala (Ala2) in receptor triggered myddosome formation model (Fig. 4D). Tested MyD88 construct and NF-κB-driven reporter were co-transfected into HEK293 cells. Wild type cells were used to allow the detection of dominant negative effect of MyD88S (a model of MyD88 isoform switching). The transfected cells were stimulated with IL-1β or TNFα to distinguish MyD88-dependent and -independent effects, respectively.

The level of reporter gene expression in IL-1β stimulated, empty vector transfected control cells reflects endogenous MyD88 signaling in wild type cells (Fig. 4A). Overexpression of MyD88L above the endogenous level has only little effect on IL-1β induced transgene expression demonstrating that the endogenous signaling is already close to saturation. MyD88S overexpression has a dominant negative effect dose dependently reducing IL-1β induced transgene expression. Also MyD88(114–118)Ala (Ala2) overexpression dose dependently reduced IL-1β induced transgene expression at the level comparable to MyD88S, demonstrating that substitution of Gln114-Leu118 in the ID of MyD88L is sufficient to mimic the dominant negative effect of MyD88S. Overexpression of randomly selected mutant, MyD88(129–133)Ala (Ala5) had no effect, again demonstrating that amino acid composition of the major part of ID is irrelevant for its function.

MyD88 independent TNFα signaling was evaluated in parallel, to control for even transduction with the reporter plasmid and for unspecific effects of overexpression of MyD88 variants on the overall cell metabolism. Neither of tested MyD88 variants had significant effect on MyD88 independent TNFα pathway, supporting the specific nature of the effects induced by tested variants within IL-1β pathway.

To further support the specific, myddosome formation related nature of the effects of tested MyD88 variants on reporter gene expression we looked at the activity of effector proteins involved in classical MyD88 signaling. The total level of cellular IRAK4, a kinase directly downstream MyD88 in myddosome assembly, has not been affected by overexpression of any tested MyD88 variants in MyD88 deficient cells (Fig. 4B). Overexpression of MyD88L induced IRAK4 phosphorylation, consistent with the known ability of MyD88L to initiate myddosome formation, recruitment and activation of IRAK4. Overexpression of MyD88(129–133)Ala (Ala5) resulted in phosphorylation of IRAK4 comparable to that induced by MyD88L demonstrating that the mutant is able to recruit and activate IRAK4 in a manner comparable to the wild type. Overexpression of MyD88S induced no IRAK4 phosphorylation consistent with the inability of this splicing variant to induce myddosome signaling. Phosphorylation of IRAK4 has neither been detected upon overexpression of MyD88(114–118)Ala (Ala2) consistent with the observation that this mutant does not sustain myddosome signaling. Moreover, unlike MyD88L and MyD88(129–133)Ala (Ala5), neither MyD88S nor MyD88(114–118)Ala (Ala2) were able to recruit IRAK4 kinase in a pulldown assay (Fig. 4C). This demonstrates that lack of phosphorylation of IRAK4 upon overexpression of the latter two variants of MyD88 is associated with compromised physical interaction of relevant MyD88 variants and IRAK4. These results collectively suggest that switching of MyD88 splicing variants mediates regulation of myddosome signaling by affecting the very initial step of myddosome complex formation—recruitment and activation of IRAK4.

IRAK1 kinase is recruited directly downstream of IRAK4 within the spatial organization of the myddosome. MyD88L overexpression resulted in phosphorylation of IRAK1 and resultant depletion of non-phosphorylated kinase (Fig. 4B), both consistent with the nucleating effect of MyD88L in myddosome formation. MyD88(129–133)Ala (Ala5) had similar effect on IRAK1 phosphorylation as the wild type MyD88L. Contrary, overexpression of MyD88S had no effect on IRAK1 phosphorylation consistent with inability of MyD88S to induce phosphorylation of IRAK4. The effect of MyD88(114–118)Ala (Ala2) overexpression on IRAK1 phosphorylation was comparable to that of MyD88S, again demonstrating that substitution of only five residues in the ID of MyD88L is sufficient to mimic the effect of physiological ID removal in MyD88S.

Overexpression of both MyD88L and MyD88(129–133)Ala (Ala5) induced phosphorylation of IκBα, the primary regulator of NF-κB (Fig. 4B), demonstrating that the signal initiated upon kinetically driven myddosome assembly, and propagated by IRAK kinase phosphorylation indeed converges at NF-κB activation. Again, overexpression of either MyD88S or MyD88(114–118)Ala (Ala2) had no effect on the phosphorylation state of IκBα, consistent with the fact that neither MyD88S nor MyD88(114–118)Ala (Ala2) induced phosphorylation of downstream IRAK kinases.

### Tyr116 is the central functional residue within the intermediate domain of MyD88L

**Fig. 5:**
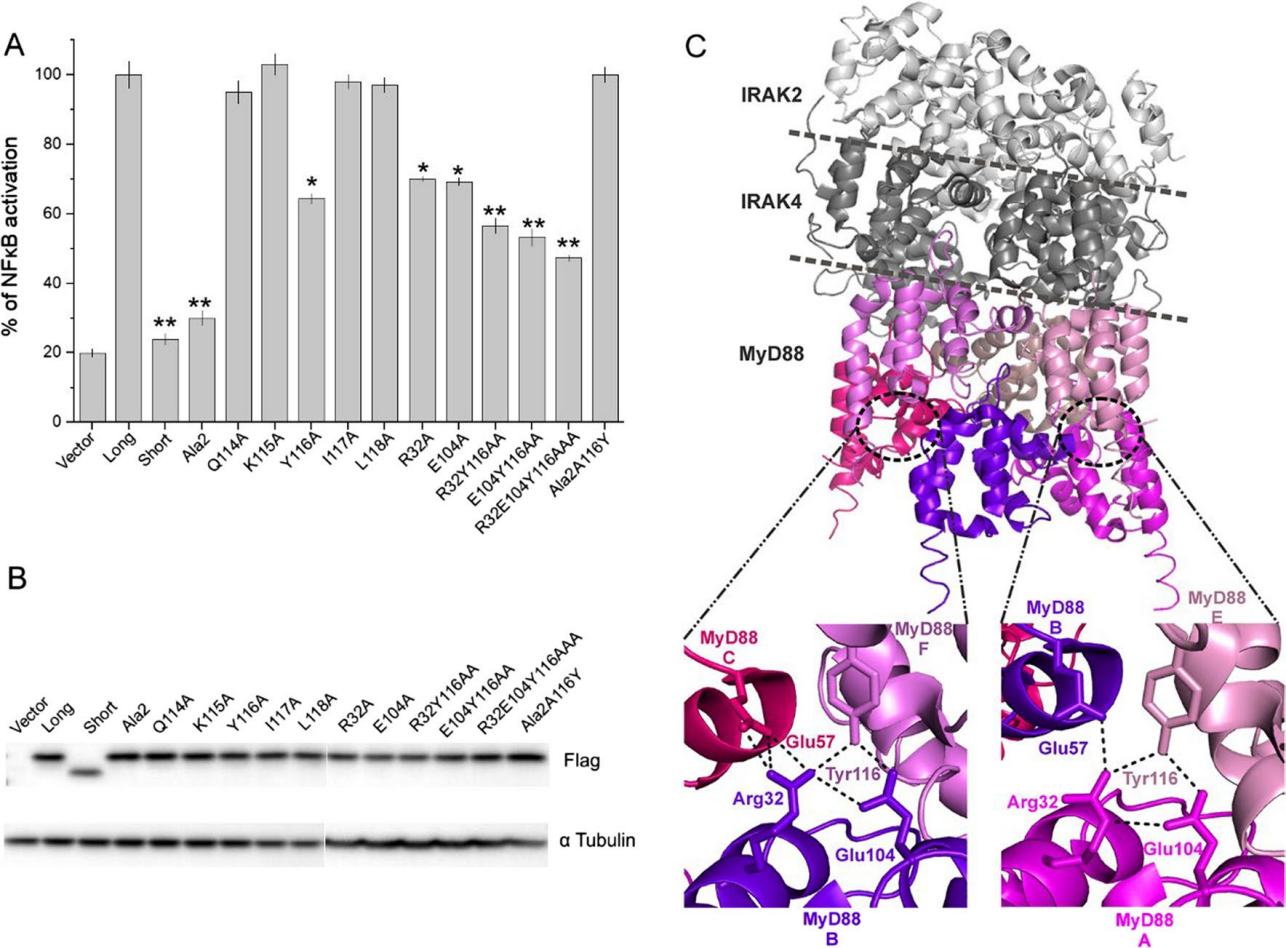
Hydrogen bond network around Tyr116 facilitates myddosome dependent signaling. **A** Induction of NF-κB-driven reporter expression in HEK293-I3A cells by overexpression of indicated MyD88 variants. Response was normalized to wild type MyD88L. Values are presented as means ± SD from triplicate samples. Statistical significance was determined by Student’s t-test (*p < 0.05, **p < 0.001). **B** Transgene expression in experiment shown in panel **A** was analyzed by Western blot. α-tubulin was used as a loading control. **C** The hierarchical myddosome assembly (PDB ID 3MOP). Intermolecular hydrogen bond network around Tyr116 mediating the homotypic interactions of adjacent MyD88 protomers are shown in close-ups

We demonstrated above that at most five out of 46 residues constituting MyD88L ID are essential for myddosome assembly. To dissect the functions of particular residues within functionally relevant Gln114-Leu118 fragment we again employed alanine scanning coupled NF-κB reporter assay. Single residue mutants MyD88(Q114A), K115A, I117A and L118A stimulated transgene expression at the level of MyD88L demonstrating that these residues are dispensable in ID function (Fig. 5A, B). Only MyD88(Y116A) resulted in significant reduction in transgene expression compared to the wild type (MyD88L), demonstrating the essential role of Y116 in mediating ID function. The effect of Y116A was less pronounced compared to that of MyD88(114–118)Ala (Ala2) suggesting that in the absence of Y116 other residues in the vicinity may partially compensate its function. However, the primary role of Y116 is corroborated by the fact that A116Y completely rescues the phenotype of MyD88(114–118)Ala (Ala2) to that of the wild type (MyD88L).

The initial eight residues of ID (Glu110-Ile117) are defined in the crystal structure of the myddosome [9] providing clues concerning the structural role of Y116. The fragment of ID defined by the electron density supports homotypic interactions of MyD88 DDs. In fact, the intermolecular interactions within ID are contributed only by Y116 participating in the network of hydrogen bonds with Arg32 and Glu104 of adjacent MyD88 DDs (Fig. 5C). To evaluate the involvement of these interactions in myddosome signaling we tested the ability of single, double and triple mutant overexpression to stimulate NF-κB driven reporter. MyD88(R32A) and E104A were compromised in their ability to activate NF-κB responsive promoter compared to MyD88L and demonstrated activity similar to MyD88(Y116A). Double mutants (Y116A, R32A) and (Y116A, E104A) were further compromised compared to the single mutants. The triple mutant was characterized by the lowest activating potential, demonstrating the importance of the hydrogen bond network around Y116 in myddosome nucleation. However, the activity of none of the mutants reached the level of MyD88(114–118)Ala (Ala2) or MyD88S again demonstrating that adjacent residues (Gln114, Lys115, Ile117 and Leu118) may partially compensate the function of the hydrogen bond network involving Y116.

### Functional ID is not essential for homotypic MyD88 interactions

**Fig. 6:**
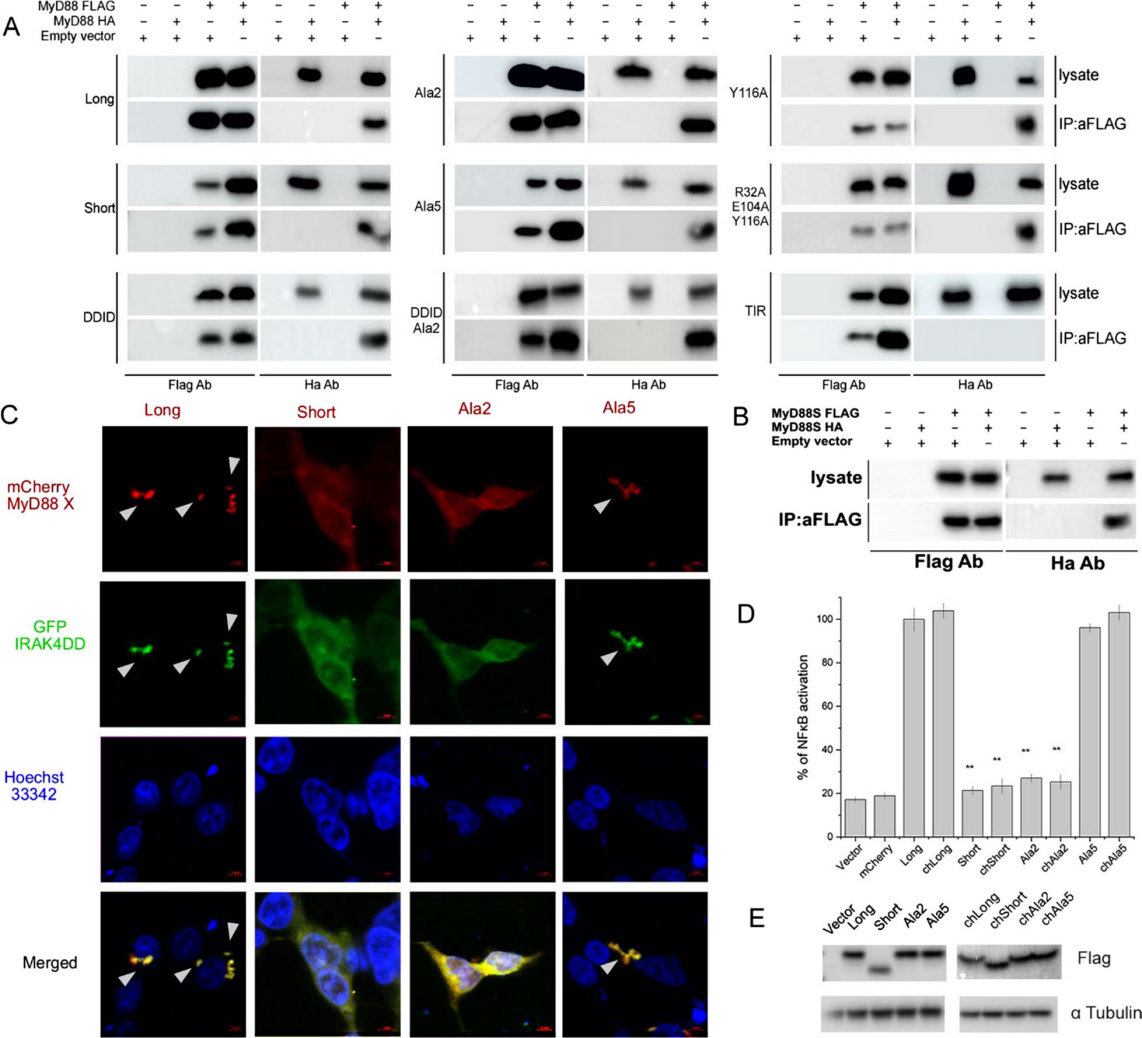
ID controls myddosome maturation. **A** Homotypic interactions of MyD88 variants. HA-tagged MyD88L was co-expressed with indicated (left) Flag-tagged MyD88 variants. Co-immunoprecipitation on anti-Flag beads was evaluated using α-HA Western blot. **B** Same as in panel **A**, but HA-tagged MyD88S was used. **C** Formation of supramolecular organizing centers (SMOCs; indicated by arrowheads) by indicated (top) MyD88 variants. mCherry labeled MyD88 variants were overexpressed in HEK293-I3A cells and imaged by confocal microscopy. Colocalization with GFP-labeled IRAK4 was evaluated. **D** mCherry fusion does not affect the NF-κB responsive promoter activating ability of overexpressed MyD88 variants. Values are presented as means ± SD from triplicate samples. Statistical significance was determined by Student’s t-test (**p < 0.001). **E** Transgene expression in experiment shown in panel **D** analyzed by Western blot. α-tubulin was used as the loading control

The dominant negative effect of MyD88 variants lacking functional ID requires homotypic interaction with MyD88L. To evaluate this requirement, HA-tagged MyD88L was co-expressed with tested Flag-tagged MyD88 variants, the putative complexes were pulled down using anti-Flag beads and analyzed for putative co-immunoprecipitation of MyD88L using α-HA. Expectedly, HA-MyD88L co-precipitated with Flag-MyD88L demonstrating that the native complex is stable at assay conditions and that tagging does not interfere with the interaction (Fig. 6A). MyD88S was able to pull down MyD88L from the cell lysate suggesting that ID is dispensable for the interaction. Correspondingly, all tested mutants within ID, regardless their ability to induce myddosome dependent signaling, were effective in pulling down MyD88L from the cell lysate (Fig. 6A), again suggesting that ID is dispensable for heterodimerization (and possibly hetero-oligomerization) of MyD88 variants. Further, isolated TIR domain was not effective in pulling down MyD88L, suggesting that homotypic TIR/TIR interactions, if relevant at all, have only transient nature and that myddosome nucleation is guided primarily by interaction of DDs. The latter conclusion is corroborated by the fact that a construct containing only DD and ID (DD-ID) was able to pull down MyD88L. Unfortunately, we were unable to obtain detectable expression of Flag- or HA- tagged DD to test its presumed ability to pull down MyD88L. However, we show that DD-ID(Ala2) pulls down MyD88L, again demonstrating that the ability to initiate myddosome nucleation is distinct from the capability to sustain signaling. A simple explanation would require MyD88S mediated termination of myddosome nuclei expansion, however Flag-MyD88S is able to pull down HA-MyD88S (Fig. 6B) demonstrating that the mechanism of signal termination is more complex.

The above findings suggest that ID is dispensable for myddosome nucleation defined as MyD88 homotypic interactions involving at least dimerization and possibly further oligomerization, which our co-immunoprecipitation assay does not allow to distinguish.

### Functional ID is essential for the formation of myddosome supramolecular organizing centre (SMOC)

MyD88 signals through nucleation of myddosome SMOC which propagates the receptor activation on the cascade of IRAK kinases. To follow SMOC formation by confocal microscopy we tagged tested variants of MyD88 with mCherry. The activity of tested variants of chMydD88 in NF-κB reporter assay was comparable to nonlabelled counterparts (Fig. 6D), demonstrating that labelling had no significant effect on signalling. Overexpression of chMyD88L in HEK293-I3A cells resulted in granular pattern of red fluorescence while mCherry showed even cytoplasmic distribution (not shown) indicating MyD88L mediated SMOC formation (Fig. 6C). C-terminal GFP tagged IRAK4 (only DD was used to avoid autophosphorylation induced dissociation of IRAK4 from the myddosome) co-localized with chMyD88L, demonstrating recruitment of the kinase to the myddosome. When mCherry-tagged MyD88S was overexpressed, uniform cytoplasmatic distribution was observed demonstrating that ID is essential for SMOC formation (Fig. 6C). Consequently, distribution of IRAK4 was also uniform (Fig. 6C) and NF-κB responsive promoter remained silent (Fig. 6D). Tested fusion protein variants had comparable expression (Fig. 6E) demonstrating that the distribution effects are concentration independent. chMyD88(114–118)Ala (chAla2) mimicked the uniform cytoplasmic distribution of MyD88S, again demonstrating the essential role of residues Gln114-Leu118 within the ID. Correspondingly, the distribution of co-expressed IRAK4 was also uniform and NF-κB responsive promoter was silent. chMyD88(129–133)Ala (chAla5) has shown granular distribution characteristic for MyD88L. IRAK4 co-localized to the granules corresponding with uncompromised activity of this mutant in induction of NF-κB responsive promoter. In fact, the activity of tested MyD88 variants in SMOC induction correlated with NF-κB activation, suggesting that SMOC is a prerequisite in myddosome signalling, and again demonstrating that Gln114-Leu118 constitute the central functional residues within ID.

**Fig. 7:**
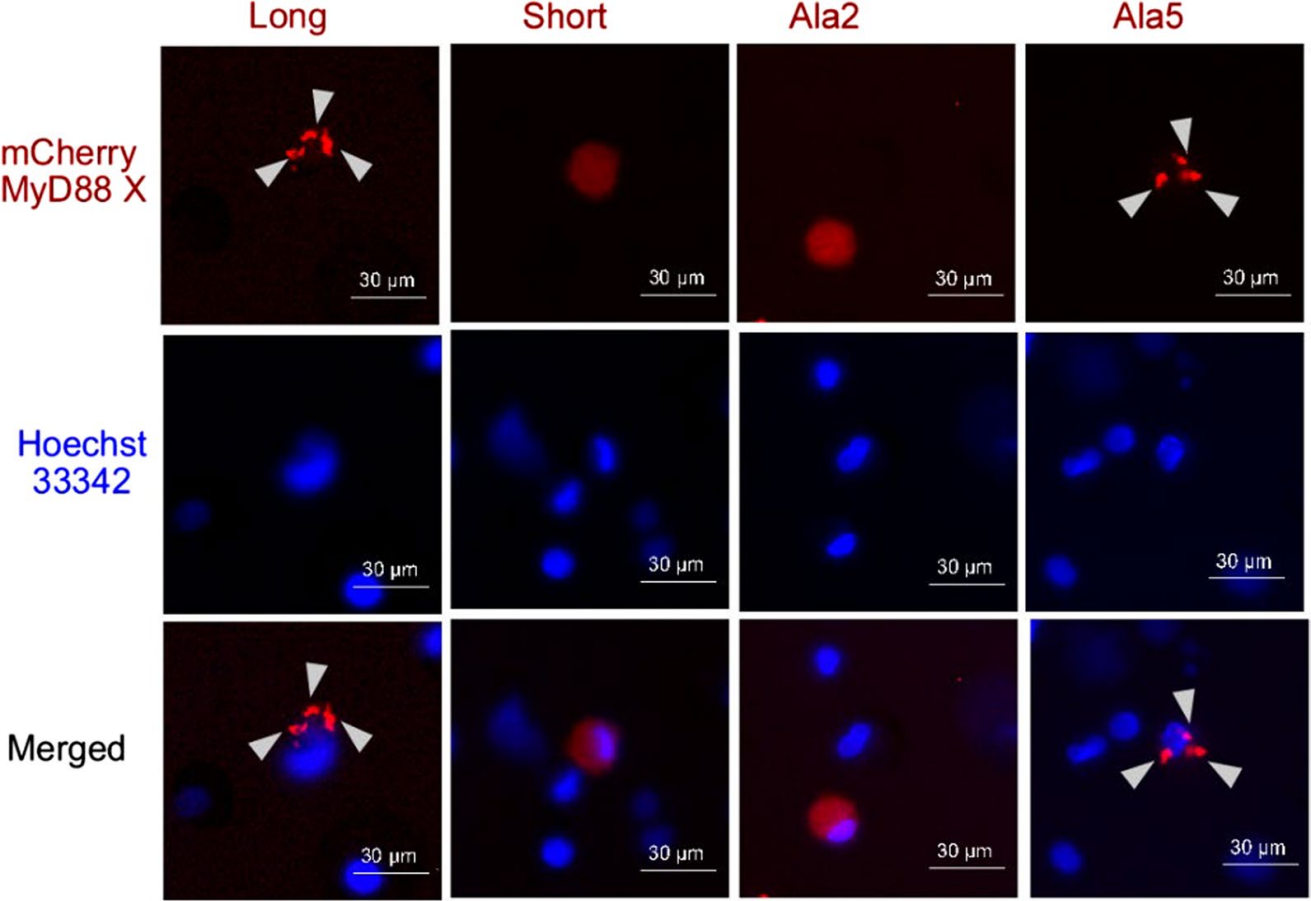
MyD88 ID is essential for myddosome formation in human primary macrophages. mCherry labeled MyD88 variants (indicated on top of the figure) were overexpressed in human monocyte-derived macrophages (hMDMs) differentiated from peripheral blood mononuclear cells (PBMCs) and imaged by fluorescence microscopy. Formation of supramolecular organizing centers (SMOCs) is indicated by arrowheads. Diffuse distribution of the transgene with no indication of SMOC formation is evident for MyD88S and Ala2

Given the limitations of HEK293-I3A cell model we verified the role of ID in myddosome nucleation using immune cells. Murine macrophage cell line RAW264.7 and primary cells, human monocyte-derived macrophages (hMDMs) differentiated from peripheral blood mononuclear cells (PBMCs) were transfected with plasmids encoding tested mCherry-labeled MyD88 variants and the distribution of the transgene was analysed by fluorescence microscopy. Expectedly, chMyD88L was characterized by granular distribution consistent with SMOC formation (for hMDMs see Fig. 7, for RAW264.7 see Additional file 1: Fig. S1A). Similarly, the distribution of chMyD88(129–133)Ala (chAla5) in immune cells was identical (granular) as that observed earlier in HEK293-I3A cells. In turn, chMyD88S and chMyD88(114–118)Ala (chAla2) both showed uniform cytoplasmic distribution suggesting impaired ability of SMOC formation. This finding again corresponded to observations in HEK293-I3A cells. Furthermore, in RAW264.7 cells, the granular distribution of MyD88 variants correlated with NF-κB activation (Additional file 1: Fig. S1B; the effect was not tested in hMDMs due to limited amount of human material). Overall, our results demonstrate that ID, and in particular amino acids 114–118 are essential for myddosome nucleation and associated signal transduction. The effect is visible irrespective the cell line used for experiments and in particular is observed in primary human immune cells, a model most physiologically relevant for investigation of Myd88 signalling.

### MyD88S stalls the growth of myddosome nuclei

**Fig. 8:**
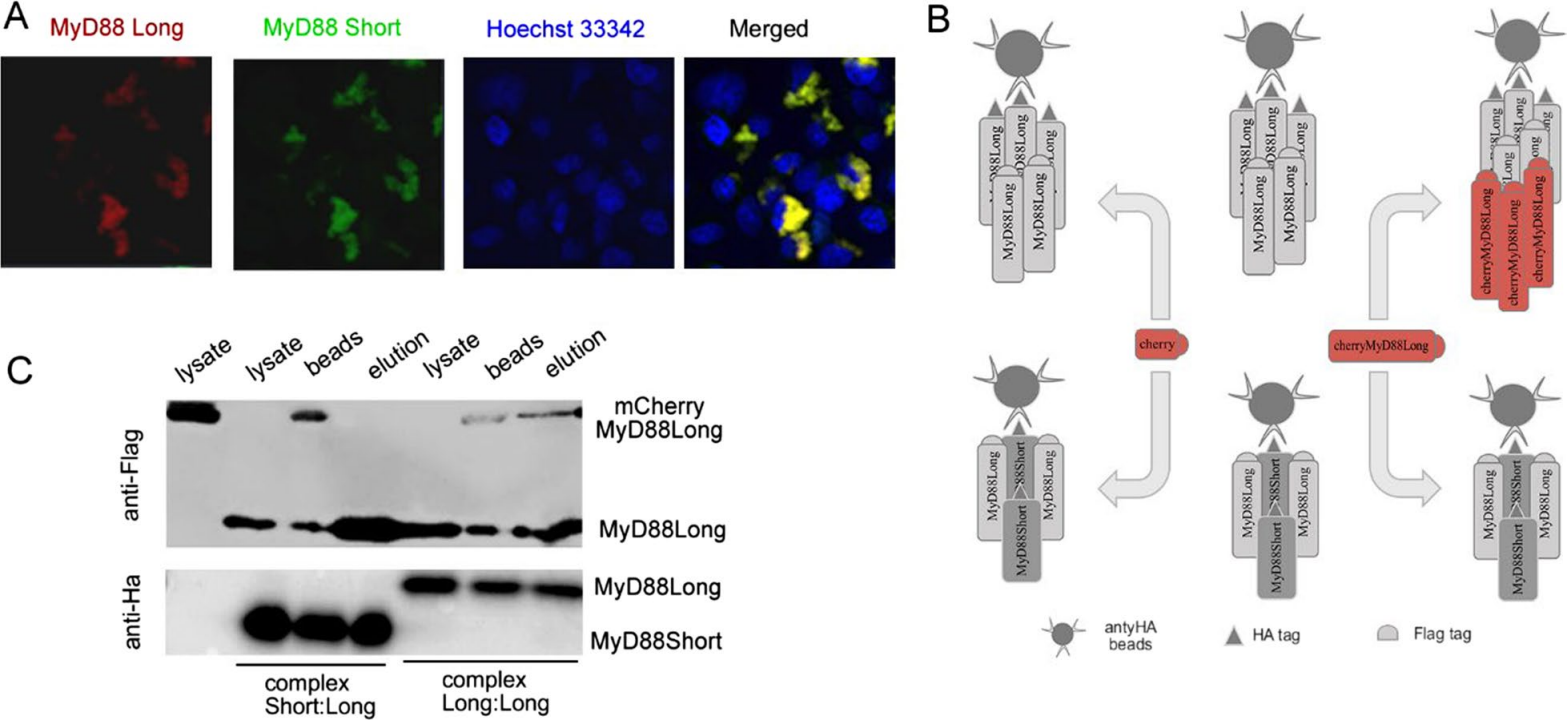
MyD88S stalls MyD88L overexpression associated myddosome SMOC formation. **A** chMyD88L overexpression associated formation of myddosome SMOC (granular distribution—refer to Fig. 6C) is hindered by simultaneous overexpression of GFP-MyD88S. Labeled MyD88 variants were overexpressed in HEK293-I3A cells and imaged by confocal microscopy. **B** Schematic representation of myddosome nuclei expansion assay. Myddosome nuclei containing HA-MyD88L and either Flag-MyD88L or Flag-MyD88S are isolated by immunoprecipitation and contacted with Flag-chMyD88L. Note that the depicted complex stoichiometries are arbitrary chosen and are not determined in the experiment. **C** MyD88L/MyD88L, but not MyD88S/MyD88L myddosome nuclei are capable of growth by incorporation of additional MyD88L molecules—Western Blot analysis of experiment schematically explained in panel **B**

The dominant negative effect of MyD88S may be mechanistically explained by an essential role of ID in myddosome nuclei expansion (oligomerization of MyD88 [8]). To test this assumption we investigated the impact of MyD88S on the formation of MyD88L overexpression associated SMOCs. When chMyD88L was co-expressed with GFP-labelled MyD88S uniform cytoplasmatic distribution of green fluorescence was observed comparable to the distribution of chMyD88S overexpressed alone, indicating that MyD88S is not tethered to MyD88L granules. In fact, uniform cytoplasmic distribution of chMyD88L red fluorescence with no indication of granulation was observed, indicating that MyD88S is not only incapable of SMOC nucleation itself, but additionally exhibits dominant negative effect over MyD88L (Fig. 8A).

We hypothesized that MyD88S incorporation into the myddosome nuclei stalls further growth of myddosome SMOC (recruitment of additional MyD88 molecules). To test this assumption we used myddosome nuclei expansion assay (Fig. 8B). Flag-MyD88L was co-expressed with either HA-MyD88L or HA-MyD88S in HEK293-I3A cells. Complexes were pulled down on α-HA magnetic beads and in each case contained both HA and Flag labelled components as indicated by Western Blot, again demonstrating that the ID is not essential for homotypic interactions of MyD88 molecules. The capability of further growth of such obtained myddosome nuclei was tested by incubation with excess of chMyD88L. Significantly, only HA-MyD88L/Flag-MyD88L, but not HA-MyD88S/Flag-MyD88L was able to recruit chMyD88L (Fig. 8C). This result directly demonstrates that incorporation of MyD88S into myddosome nuclei stalls the growth of myddosome SMOC providing the rationale for the dominant negative effect of MyD88S in myddosome mediated signalling.

## Discussion

The universal adaptor molecule, MyD88, propagates major activation signals driving the innate immune response. In turn, its alternative splicing variant, MyD88S quenches the response upon prolonged exposure to stimuli [30]. In this study we provided mechanistic insight into the above functional observations.

MyD88 is a modular protein, where DD acts as a multimerization platform and a bait for cytoplasmic IRAK kinases, while TIR domain provides interaction with membrane receptors. Thereby, MyD88 bridges the signal recognized at the membrane with intracellular effectors. DD and TIR domain are separated by 46 amino-acid long ID encoded by exon 3, the only fragment missing in MyD88S. As such, ID was attributed a role of a steric spacer separating the functions of DD and TIR. Here we demonstrated that ID is not only a spacer, but contains functional residues. Alanine scanning revealed that substitution of residues Gln114-Leu118 mimics the dominant negative effect of MyD88S. In fact, alanine substitution of a single residue, Tyr116, was sufficient. Careful analysis of available structural data on the myddosome [9, 17] suggests a mechanistic explanation. The C-terminal helix of MyD88 DD is longer compared to classical DD fold and the helix extends over the N-terminal part of ID, beyond the globular fold of the DD. Within the extension, Tyr116 provides homotypic interactions with adjacent DDs, a feature which importance was overlooked in prior analysis [9], likely because of seemingly more pronounced, extensive homotypic interface of the core DDs. Here, we demonstrated that the hydrogen bond network connecting Tyr116, Arg32 and Glu104 of adjacent MyD88 subunits of the myddosome is central to signal propagation. Substitution of any of those residues with alanine compromised MyD88-mediated signal transduction. The importance of Tyr116 in MyD88 signaling is supported by in vivo data. Random germline mutagenesis identified Y116C to diminish the response to pathogen-associated stimuli in mice [31].

Inability of certain MyD88 mutants and variants to sustain signaling does not explain the dominant negative effect of those variants in presence of MyD88L. Upon interaction with activated membrane receptors, MyD88L seeds the myddosome supramolecular organizing center (SMOC) where increased molecular crowding drives signal amplification. Prior to this study it was unclear how does MyD88S quench the potentially damaging inflammatory backlash. It has been demonstrated that unlike MyD88L, MyD88S is unable to bind IRAK4 [16], but this observation does not explain the dominant negative effect of MyD88S in the presence of MyD88L. Such effect would require incorporation of the former isoform into the myddosome resulting in termination of its assembly. We have shown via direct co-immunoprecipitation that indeed MyD88L interacts with MyD88S (as well as all the signaling deficient mutants evaluated in this study) and that the interaction is mediated by DD and not TIR domain. This last observation is consistent with the fact that mammalian TIR domains form only transient intermolecular interactions [32]. One could speculate that MyD88L/MyD88S interaction terminates myddosome outgrowth and indeed co-expression of GFP-MyD88S hinders MyD88L associated SMOC formation observed as a speckled pattern of mCherry-MyD88L in confocal microscopy. Nonetheless, questions remain: how does MyD88S terminates the singling while it is able to recruit MyD88L and why MyD88S hinders SMOC formation if it recruits both MyD88L and MydD88S as shown by co-immunoprecipitation. To answer these questions we had to overcome the inherent limitation of simple co-IP assay, that is its insensitivity to stoichiometry. Using myddosome nuclei expansion assay we demonstrated that (MyD88L)_n_ nuclei are easily extended with additional MyD88L molecules while MyD88L_x_/MyD88S_y_ nuclei are unable to incorporate additional MyD88L molecules. This experiment demonstrated that MyD88S exhibits a capping effect. Mixed dimers (or possibly small multimers) of MyD88L and MyD88S are observed in the experiment, however MyD88S containing nuclei are incapable of further outgrowth thus terminating the signaling.

Interestingly, a comparable scenario was adapted by speckle-type POZ protein (SPOP) to quench Toll-like receptor induced response. SPOP is a nuclear ubiquitin ligase adapter protein, however TLR stimulation leads to its nucleus-cytoplasm translocation and binding to MyD88 which blocks NF-κB activation, an effect not involving ubiquitination, but rather inhibition of mature myddosome formation [33].

Overall, our functional data and prior structural information [9, 17] allow to propose a consistent model of MyD88S mediated termination of myddosome signaling. By virtue of homotypic DD interactions, MyD88 nuclei (dimers/oligomers) recruit further MyD88 molecules to form helical assemblies capable only of unidirectional extension. Heterotypic DD interactions recruit IRAK effector kinases which propagate the signal. Incorporation of MyD88S into MyD88 nuclei precludes further homotypic and heterotypic DD interactions thus terminating both the myddosome SMOC assembly and signal propagation.

## Conclusions

We provided the mechanistic understanding of the role of Myd88S in restricting the nucleation of the myddosome complex and associated signal transduction. We demonstrated that the largely unstructured ID of Myd88 is essential for productive nucleation of the myddosome and that Tyr116 is the only functionally vital residue within ID. Dominant negative splicing variant of Myd88, MyD88S binds the early myddosome nucleation intermediates and exerts a capping effect restricting incorporation of further components thus terminating the signal transduction.

## Materials and methods

### Cell culture

Human HEK293 and RAW264.7 cell lines were obtained from the European Collection of Cell Cultures. MyD88 KO HEK293 cell line (HEK293-I3A) was a kind gift from R. Jerala (Department of Biotechnology, National Institute of Chemistry, Ljubljana, Slovenia). Cells were cultured in minimal DMEM medium (Invitrogen) supplemented with 10% fetal bovine serum (Lonza). Cells were maintained at 37 °C in a humidified atmosphere containing 5% CO_2_.

Human monocyte-derived macrophages (hMDMs) differentiated from peripheral blood mononuclear cells (PBMCs) were isolated as described previously [34]. Briefly, PBMCs were obtained from human blood using lymphocyte separation medium (Pancoll, PanBiotech, Germany) density gradient yielding a fraction enriched in monocytes. Cells were seeded on 24-well plates (Corning, USA) in RPMI-1640 medium supplemented with 10% heat-inactivated autologous human plasma and 50 μg/ml gentamicin. After 24 h, non-adherent PBMCs were removed by washing with complete medium, and adherent cells were cultured for 7 days under standard conditions (37 °C, 5% CO_2_) with fresh medium changed every 2 days.

Blood was obtained from the Red Cross (Krakow, Poland), which de-identifies blood materials as relevant for human subject confidentiality assurance. As such, this study adheres to relevant exclusions from human subject approval.

### Plasmids

DNA encoding full length MyD88 (MyD88L), MyD88 alanine mutants (Ala1-9), and IRAK death domain (Arg20-Ala104) with N-FLAG (MDYKDDDDK) or N-HA (MYPYDVPDYS) tags was synthesized by Genscript and cloned into pcDNA3.1 for overexpression. Other constructs were obtained by site-directed mutagenesis using the method described by Edelheit and colleagues [35] and primers summarized in Additional file 1: Table S1. Fluorescent protein fusions were prepared by appending genes encoding mCherry or Turbo GFP using restriction free cloning [36]. NF-κB driven firefly luciferase and *Renilla* luciferase constructs were obtained from Promega.

### NF-κB reporter assay

HEK293 and HEK293-I3A cells were seeded in 24-well plates at the density of 2 × 10^5^ cells/well. 24 h later the cells were transfected with tested plasmid DNA using the Lipofectamine 2000 (Invitrogen, USA). 0.5 µg of plasmid DNA per well was used, including 0.05 µg of tested MyD88 variant encoding plasmid or an empty vector control and 0.45 µg of the reporter vectors (0.4 µg of firefly and 0.05 µg of *Renilla* luciferase encoding plasmids). 24 h after transfection the cells were either left untreated or were stimulated for 6 h with 10 ng/ml IL-1β (PromoKine) or 100 ng/ml TNFα (PromoKine). 48 h post-transfection the cells were lysed using Passive Lysis Buffer (Promega, USA), and firefly and *Renilla* luciferase activities were determined using Dual Luciferase Reporter Assay System (Promega, USA). Data are presented as relative luciferase activities (firefly / *Renilla*) and expressed as a percentage of activity of full length protein (MyD88L). Mean ± SD data of triplicate samples and representative of at least three independent experiments is shown.

### Analysis of expression and phosphorylation profiles

HEK293-I3A cells were seeded in 24-well plates at the density of 2 × 10^5^ cell/well. 24 h later the cells were transfected with 0.5 µg of plasmid DNA using Lipofectamine 2000 (Invitrogen, USA). 48 h later cells were lysed in RIPA buffer containing protease inhibitors (Sigma-Aldrich) and phosphatase inhibitors (Calbiochem). Total cell proteins (10 µg) were separated by 12% SDS-PAGE and transferred to PVDF membrane (ThermoScientific). MyD88 variants were detected using anti-FLAG monoclonal antibody (Sigma-Aldrich; F3165). Phosphorylated and unphosphorylated proteins were detected using rabbit mAb anti-IκB (Abcam; E130), mAb anti-phosphoIκB (pSer32) (Abcam; EPR3148), pAb anti-IRAK4 (Cell Signaling; PA5-20,018), mAb anti-phosphoIRAK4 (pThr345/pSer346) (Cell Signaling; D6D7), mouse mAb anti-alpha tubulin (Abcam; DM1A). HRP-conjugated polyclonal goat anti-mouse IgG (Santa Cruz Biotechnology; SC-2005) and polyclonal goat anti-rabbit IgG (Abcam; AB6721) were used as secondary antibodies.

### Co-immunoprecipitation

HEK293-I3A cells were co-transfected with plasmids encoding N-terminal HA-MyD88L (1 µg) and tested N-terminal Flag-MyD88 variants (1 µg). 48 h later cells were lysed by sonication in lysis buffer (50 mM Tris–HCl pH 7.4, 0,1% CHAPS, 150 mM NaCl) with protease inhibitor cocktail (Sigma-Aldrich). The lysates were clarified by centrifugation. 0.3 mg of total cell proteins was incubated overnight with anti-FLAG beads (Sigma-Aldrich) at 4 °C with gentle agitation. The beads were washed 5 times with lysis buffer to remove impurities, the complexes were eluted with 0.1 mM glycine pH 3.0. The eluted fraction was separated on 12% SDS-PAGE and analyzed by Western blot using anti-FLAG (Sigma Aldrich; F3165), anti-HA (Cell signaling; C29F4) or anti-IRAK4 (Cell Signaling; PA5-20,018) and relevant HRP-conjugated secondary antibodies.

### Myddosome nuclei expansion assay

HEK293-I3A cells were transfected with a plasmid expressing Flag-mCherry-MyD88L or co-transfected with plasmids expressing Flag-MyD88L and either HA-MyD88L or HA-MyD88S. After 48 h, cells were lysed by sonication in lysis buffer and the lysates were clarified by centrifugation. Lysates from co-transfection (0.3 mg of total cell protein) were incubated with anti-HA beads (Thermo Scientific) for an hour at RT. Lysates containing Flag-mCherry-MyD88L were added (0.3 mg of total cell proteins) and incubated overnight at 4 °C with gentle agitation. The samples were washed and analyzed by Western blot (anti-FLAG and anti-HA) exactly as described for co-immunopreciptiation.

### NMR

2.2 mg of synthetic peptide corresponding to residues Glu110-Gly155 of human MyD88L (GenBank no. U70451) was dissolved in 200 µl of H_2_O and 20 µl of D_2_O was added to the sample to provide the lock signal and ^1^H NMR spectrum was acquired. The deuterium oxide (2000 µl) was added and ^1^H NMR spectrum was acquired. Spectra were recorded at 300 K using a Bruker Avance 600 MHz spectrometer.

### Size exclusion chromatography

Synthetic peptide corresponding to ID of MyD88L (1 mg/ml; 100 µl) was analyzed by size exclusion chromatography in PBS with or without 9 M urea using Superdex 200 GL column (GE Healthcare Life Sciences) at 0.7 ml/min. Partition coefficients K_AV_ and Stokes radii (R_S_) were calculated using calibrated retention coefficients as described elsewhere [37].

### Circular dichroism

CD spectra were recorded in PBS with or without 9 M urea using J-710 spectropolarimeter (Jasco) in the range of 190–250 nm and averaged over five acquisitions. Recorded data were converted to molar residual ellipticity units as described elsewhere [38]. The secondary structure composition was estimated using CDPro spectrum decomposition software [39] running CONTINLL algorithm on SDP48 reference spectrum data set.

### Secondary structure prediction

The structure of ID (Glu110-Gly155) of MyD88 was predicted using iterative threading assembly refinement as implemented in I-TASSER server and described elsewhere [27–29]. The model with the highest confidence was selected for discussion.

### Confocal microscopy

HEK293-I3A and RAW264.7 cells were seeded in 35 mm glass-bottom dish at density 5 × 10^5^ cell/well. 24 h later the cells were transfected with tested plasmids using Lipofectamine 2000 (Invitrogen, USA). 24 h after transfection the cells were washed with 1 ml PBS and the nuclei were stained with Hoechst 33,258 (ThermoScientific) for 10 min at 37 ºC. The cells were washed with PBS, fixed with 4% PFA and visualized using Zeiss LSM880 scanning microscope (mCherry: ex. 543 nm, em. 600–680 nm; GFP: ex. 488 nm, em. 500–540 nm; Hoechst 33258: ex. 405 nm, em. 420–460 nm).

### Fluorescence microscopy

Human macrophages seeded in 24-well plate at density 3 × 10^5^ cell/well were transfected with tested plasmids using Lipofectamine 2000 (Invitrogen, USA). 24 h after transfection the nuclei were stained with Hoechst 33,342 (ThermoScientific) for 10 min at 37 ºC and the culture medium was changed. The cells were visualized using Leica DMi8 fluorescent microscope (mCherry: ex. 543 nm, em. 600–680 nm; Hoechst 33342: ex. 350 nm, em. 460–490 nm).

### Statistical analysis

GraphPad Prism 5.0 was used for statistical analysis. Significance of differences between treatment groups was determined using Student’s t-test. p < 0.05 was set as the threshold for significance (*p < 0.05, **p < 0.001). Bar charts show means ± SDs of three independent experiments.

## Supplementary Information

**Additional file 1. Supplementary Figure S1**. Myd88 ID is essential for nucleation of myddosome supramolecular organizing center (SMOC) in murine macrophage cell line RAW264.7. (A) Indicated mCherry labeled MyD88 variants (top) were overexpressed in RAW264.7 cells and imaged by confocal microscopy. Myddosome nucleation is indicated by arrowheads. (B) Plasmids overexpressing indicated MyD88 variants were transfected into RAW264.7 together with plasmids encoding NF-κB-responsive promoter driven luciferase reporter gene. Induction of reporter expression was monitored. Response was normalized to wild type MyD88L. Values are presented as means±SD from triplicate samples. Statistical significance was determined by Student’s t-test (*p<0.05, **p<0.001). Supplementary Table S1. Primers and constructs used in the study.

## Data Availability

All other data supporting the findings of this study are available from the corresponding authors upon reasonable request. Source data are provided with this paper.

## Abbreviations

DD: Death domain
dsRNA: Double-stranded RNA
ID: Intermediate domain
IL-1: Interleuin-1
IL-1R: IL-1 receptor
IRAK: Interleukin-1 receptor associated kinase
MyD88: Myeloid differentiation primary response 88
NF-κB: Nuclear factor kappa-light-chain-enhancer of activated B cells
PDB: Protein Data Bank
PRR: Pattern recognition receptor
SMOC: Supramolecular organizing center
ssRNA: Single-stranded RNA
TIR: Toll-interleukin-1 receptor homology domain
TLR: Toll-like receptor

## References

[1] Takeuchi O, Akira S (2010). Pattern recognition receptors and inflammation. Cell.

[2] Liu J, Cao X (2016). Cellular and molecular regulation of innate inflammatory responses. Cell Mol Immunol.

[3] Tsan M (2006). Toll-like receptors, inflammation and cancer. Semin Cancer Biol.

[4] Kumar H, Kawai T, Akira S (2009). Pathogen recognition in the innate immune response. Biochem J.

[5] Gao D, Li W (2017). Structures and recognition modes of Toll-like receptors. Proteins Struct Funct Bioinforma..

[6] O’Neill LAJ, Bowie AG (2007). The family of five: TIR-domain-containing adaptors in Toll-like receptor signalling. Nat Rev Immunol.

[7] Deguine J, Barton GM (2014). MyD88: a central player in innate immune signaling. F1000Prime Rep.

[8] Motshwene PG, Moncrieffe MC, Grossmann JG, Kao C, Ayaluru M, Sandercock AM, Robinson CV, Latz E, Gay NJ (2009). An oligomeric signaling platform formed by the Toll-like receptor signal transducers MyD88 and IRAK-4. J Biol Chem.

[9] Lin S-C, Lo Y-C, Wu H (2010). Helical assembly in the MyD88–IRAK4–IRAK2 complex in TLR/IL-1R signalling. Nature.

[10] Latty SL, Sakai J, Hopkins L, Verstak B, Paramo T, Berglund NA, Cammarota E, Cicuta P, Gay NJ, Bond PJ, Klenerman D, Bryant CE (2018). Activation of Toll-like receptors nucleates assembly of the MyDDosome signaling hub. Elife..

[11] Wells CA, Chalk AM, Forrest A, Taylor D, Waddell N, Schroder K, Himes SR, Faulkner G, Lo S, Kasukawa T, Kawaji H, Kai C, Kawai J, Katayama S, Carninci P, Hayashizaki Y, Hume DA, Grimmond SM (2006). Alternate transcription of the Toll-like receptor signaling cascade. Genome Biol.

[12] Hardiman G, Jenkins NA, Copeland NG, Gilbert DJ, Garcia DK, Naylor SL, Kastelein RA, Bazan JF (1997). Genetic structure and chromosomal mapping of MyD88. Genomics.

[13] Bonnert TP, Garka KE, Parnet P, Sonoda G, Testa JR, Sims JE (1997). The cloning and characterization of human MyD88: a member of an IL-1 receptor related family. FEBS Lett.

[14] Vickers TA, Zhang H, Graham MJ, Lemonidis KM, Zhao C, Dean NM (2006). Modification of MyD88 mRNA splicing and inhibition of IL-1beta signaling in cell culture and in mice with a 2’-O-methoxyethyl-modified oligonucleotide. J Immunol..

[15] Janssens S, Burns K, Tschopp J, Beyaert R (2002). Regulation of interleukin-1- and lipopolysaccharide-induced NF-kappaB activation by alternative splicing of MyD88. Curr Biol.

[16] Burns K, Janssens S, Brissoni B, Olivos N, Beyaert R, Tschopp J (2003). Inhibition of interleukin 1 receptor/Toll-like receptor signaling through the alternatively spliced, short form of MyD88 is due to its failure to recruit IRAK-4. J Exp Med.

[17] Moncrieffe MC, Bollschweiler D, Li B, Penczek PA, Hopkins L, Bryant CE, Klenerman D, Gay NJ (2020). MyD88 death-domain oligomerization determines myddosome architecture: implications for toll-like receptor signaling. Structure.

[18] Hartman ZC, Osada T, Glass O, Yang XY, Lei GJ, Lyerly HK, Clay TM (2010). Ligand-independent toll-like receptor signals generated by ectopic overexpression of MyD88 generate local and systemic antitumor immunity. Cancer Res.

[19] Loiarro M, Sette C, Gallo G, Ciacci A, Fantò N, Mastroianni D, Carminati P, Ruggiero V (2005). Peptide-mediated interference of TIR domain dimerization in MyD88 inhibits interleukin-1-dependent activation of NF-{kappa}B. J Biol Chem.

[20] Li X, Commane M, Burns C, Vithalani K, Cao Z, Stark GR (1999). Mutant cells that do not respond to interleukin-1 (IL-1) reveal a novel role for IL-1 receptor-associated kinase. Mol Cell Biol.

[21] Ngo VN, Young RM, Schmitz R, Jhavar S, Xiao W, Lim K-H, Kohlhammer H, Xu W, Yang Y, Zhao H, Shaffer AL, Romesser P, Wright G, Powell J, Rosenwald A, Muller-Hermelink HK, Ott G, Gascoyne RD, Connors JM, Rimsza LM, Campo E, Jaffe ES, Delabie J, Smeland EB, Fisher RI, Braziel RM, Tubbs RR, Cook JR, Weisenburger DD, Chan WC, Staudt LM (2011). Oncogenically active MyD88 mutations in human lymphoma. Nature..

[22] Avbelj M, Wolz O-O, Fekonja O, Benčina M, Repič M, Mavri J, Krüger J, Schärfe C, Delmiro Garcia M, Panter G, Kohlbacher O, Weber ANR, Jerala R (2014). Activation of lymphoma-associated MyD88 mutations via allostery-induced TIR-domain oligomerization. Blood..

[23] George J, Motshwene PG, Wang H, Kubarenko AV, Rautanen A, Mills TC, Hill AVS, Gay NJ, Weber ANR (2011). Two human MYD88 variants, S34Y and R98C, interfere with MyD88-IRAK4-myddosome assembly. J Biol Chem..

[24] Medzhitov R, Preston-Hurlburt P, Kopp E, Stadlen A, Chen C, Ghosh S, Janeway CA (1998). MyD88 is an adaptor protein in the hToll/IL-1 receptor family signaling pathways. Mol Cell.

[25] Narayanan KB, Park HH (2015). Toll/interleukin-1 receptor (TIR) domain-mediated cellular signaling pathways. Apoptosis.

[26] Fekonja O, Avbelj M, Jerala R (2013). Suppression of TLR signaling by targeting TIR domain-containing proteins. Curr Protein Pept Sci.

[27] Zhang Y (2008). I-TASSER server for protein 3D structure prediction. BMC Bioinform.

[28] Roy A, Kucukural A, Zhang Y (2010). I-TASSER: a unified platform for automated protein structure and functionprediction. Nat Protoc.

[29] Yang J, Yan R, Roy A, Xu D, Poisson J, Zhang Y (2015). The I-TASSER Suite: protein structure and function prediction. Nat Methods.

[30] Burns K, Martinon F, Esslinger C, Pahl H, Schneider P, Bodmer JL, Di Marco F, French L, Tschopp J (1998). MyD88, an adapter protein involved in interleukin-1 signaling. J Biol Chem.

[31] Jiang Z, Georgel P, Li C, Choe J, Crozat K, Rutschmann S, Du X, Bigby T, Mudd S, Sovath S, Wilson IA, Olson A, Beutler B (2006). Details of Toll-like receptor:adapter interaction revealed by germ-line mutagenesis. Proc Natl Acad Sci.

[32] Nimma S, Ve T, Williams SJ, Kobe B (2017). Towards the structure of the TIR-domain signalosome. Curr Opin Struct Biol.

[33] Hu Y-H, Wang Y, Wang F, Dong Y-M, Jiang W-L, Wang Y-P, Zhong X, Ma L-X (2020). SPOP negatively regulates Toll-like receptor-induced inflammation by disrupting MyD88 self-association. Cell Mol Immunol.

[34] Blazusiak E, Florczyk D, Jura J, Potempa J, Koziel J (2013). Differential regulation by Toll-like receptor agonists reveals that MCPIP1 is the potent regulator of innate immunity in bacterial and viral infections. J Innate Immun.

[35] Edelheit O, Hanukoglu A, Hanukoglu I (2009). Simple and efficient site-directed mutagenesis using two single-primer reactions in parallel to generate mutants for protein structure-function studies. BMC Biotechnol.

[36] Bond SR, Naus CC (2012). RF-Cloning.org: an online tool for the design of restriction-free cloning projects. Nucleic Acids Res.

[37] Górecki A, Bonarek P, Górka AK, Figiel M, Wilamowski M, Dziedzicka-Wasylewska M (2015). Intrinsic disorder of human Yin Yang 1 protein. Proteins Struct Funct Bioinform.

[38] Kelly SM, Jess TJ, Price NC (2005). How to study proteins by circular dichroism. Biochim Biophys Acta Proteins Proteomics.

[39] Sreerama N, Woody RW (2000). Estimation of protein secondary structure from circular dichroism spectra: comparison of CONTIN, SELCON, and CDSSTR methods with an expanded reference set. Anal Biochem.

