# Supplementary material for: Mechanism of MyD88S mediated signal termination
Source: Cell Commun Signal. 2022 Jan 20;20:10. doi: 10.1186/s12964-021-00811-1 (PMC8772076; doi:10.1186/s12964-021-00811-1)
Supplement: Supplementary file 2 — Additional file 1. Supplementary Figure S1. Myd88 ID is essential for nucleation of myddosome supramolecular organizing center (SMOC) in murine macrophage cell line RAW264.7. (A) Indicated mCherry labeled MyD88 variants (top) were overexpressed in RAW264.7 cells and imaged by confocal microscopy. Myddosome nucleation is indicated by arrowheads. (B) Plasmids overexpressing indicated MyD88 variants were transfected into RAW264.7 together with plasmids encoding NF-κB-responsive promoter driven luciferase reporter gene. Induction of reporter expression was monitored. Response was normalized to wild type MyD88L. Values are presented as means±SD from triplicate samples. Statistical significance was determined by Student’s t-test (*p<0.05, **p<0.001). Supplementary Table S1. Primers and constructs used in the study. [file 12964_2021_811_MOESM2_ESM.docx]

Mechanism of MyD88S mediated signal termination

Katarzyna Pustelny, Katarzyna Kuska, Andrzej Gorecki, Bogdan Musielak, Ewelina Dobosz, Benedykt Wladyka, Joanna Koziel, Anna Czarna, Tad Holak and Grzegorz Dubin

SUPPLEMENTARY INFORMATION

Supplementary Figure S1

Supplementary Table S1


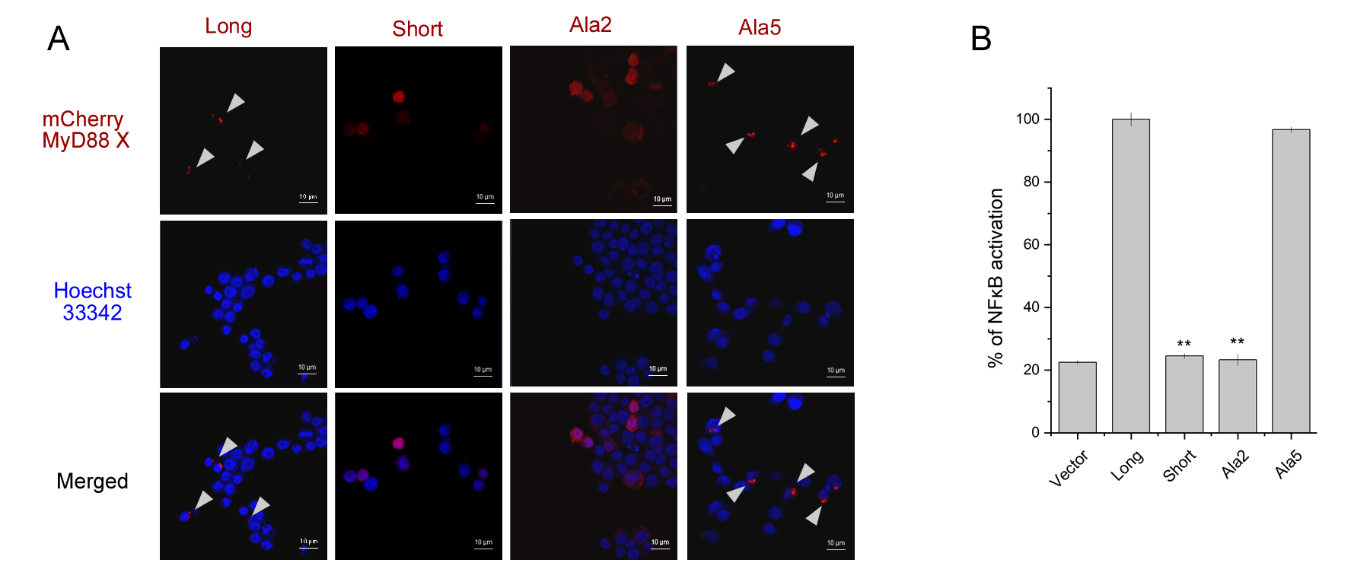


**Supplementary Figure S1. Myd88 ID is essential for nucleation of myddosome supramolecular organizing center (SMOC) in murine macrophage cell line** **RAW264.7.** (A) Indicated mCherry labeled MyD88 variants (top) were overexpressed in RAW264.7 cells and imaged by confocal microscopy. Myddosome nucleation is indicated by arrowheads. (B) Plasmids overexpressing indicated MyD88 variants were transfected into RAW264.7 together with plasmids encoding NF-κB-responsive promoter driven luciferase reporter gene. Induction of reporter expression was monitored. Response was normalized to wild type MyD88L. Values are presented as means±SD from triplicate samples. Statistical significance was determined by Student’s t-test (*p<0.05, **p<0.001).

**Supplementary Table S1. Primers and constructs used in the study.**

| **Expression construct** | **Abbreviation** | **Primers** | **Primer sequence** | **Comment** |  |
| --- | --- | --- | --- | --- | --- |
| MyD88 L | Long | n.a. | construct obtained from GenScript | expression of full length protein MyD88L |  |
| MyD88 S | Short | short_F | CTGGAGCTGGGACCCAGCATTGGGCATATGCCTGAG CGTTTC | expression of MyD88 protein lacking ID (MyD88S) |  |
|  |  | short_R | GAAACGCTCAGGCATATGCCCAATGCTGGGTCCCAGCTCCAG |  |  |
| MyD88 DD | DD | DD_F | GACGACGTGCTGCTGGAGCTGGGACCCAGCATTTAG | expression of isolated DD of MyD88 |  |
|  |  | DD_R | CTAAATGCTGGGTCCCAGCTCCAGCAGCACGTCGTC |  |  |
| MyD88 DDID | DDID | DDID_F | ATCACCACACTTGATGACCCCCTGGGGCATTAG | expression of DD with ID of MyD88 |  |
|  |  | DDID_R | CTAATGCCCCAGGGGGTCATCAAGTGTGGTGAT |  |  |
| MyD88 IDTIR | IDTIR | IDTIR_F | ATGGACTACAAAGACGATGACGACAAGGAGGAGGATTGCCAAAAG | expression of ID with TIR domain of MyD88 |  |
|  |  | IDTIR_R | CTTTTGGCAATCCTCCTCCTTGTCGTCATCGTCTTTGTAGTCCAT |  |  |
| MyD88 TIR | TIR | TIR_F | ATGGACTACAAAGACGATGACGACAAGATGCCTGAGCGTTTCGATG | expression of isolated TIR domain of MyD88 |  |
|  |  | TIR_R | CATCGAAACGCTCAGGCATCTTGTCGTCATCGTCTTTGTAGTCCAT |  |  |
| MyD88 Ala1 | Ala1 | n.a. | construct obtained from GenScript | expression of MyD88 mutant where residues E110 to C113 are substituted with alanine residues |  |
|  |  |  |  |  |  |
| MyD88 Ala2 | Ala2 | n.a. | construct obtained from GenScript | expression of MyD88 mutant where residues Q114 to L118 are substituted with alanine residues |  |
|  |  |  |  |  |  |
| MyD88 Ala3 | Ala3 | n.a. | construct obtained from GenScript | expression of MyD88 mutant where residues K119 to E123 are substituted with alanine residues |  |
|  |  |  |  |  |  |
| MyD88 Ala4 | Ala4 | n.a. | construct obtained from GenScript | expression of MyD88 mutant where residues E124 to P128 are substituted with alanine residues |  |
|  |  |  |  |  |  |
| MyD88 Ala5 | Ala5 | n.a. | construct obtained from GenScript | expression of MyD88 mutant where residues L129 to A133 are substituted with alanine residues |  |
|  |  |  |  |  |  |
| MyD88 Ala6 | Ala6 | n.a. | construct obtained from GenScript | expression of MyD88 mutant where residues V134 to V138 are substituted with alanine residues |  |
|  |  |  |  |  |  |
| MyD88 Ala7 | Ala7 | n.a. | construct obtained from GenScript | expression of MyD88 mutant where residues P139 to E143 are substituted with alanine residues |  |
|  |  |  |  |  |  |
| MyD88 Ala8 | Ala8 | n.a. | construct obtained from GenScript | expression of MyD88 mutant where residues L144 to T148 are substituted with alanine residues |  |
|  |  |  |  |  |  |
| MyD88 Ala9 | Ala9 | n.a. | construct obtained from GenScript | expression of MyD88 mutant where residues T149 to G155 are substituted with alanine residues |  |
|  |  |  |  |  |  |
| MyD88 ΔAla2 | ΔAla2 | Ala2_F | AGCATTGAGGAGGATTGCAAGCAGCAGCAGGAGGAG | expression of MyD88 mutant where residues Q114 to L118 are deleted |  |
|  |  | Ala2_R | CTCCTCCTGCTGCTGCTTGCAATCCTCCTCAATGCT |  |  |
| MyD88 Q114A | Q114A | Q114A_F | CATTGAGGAGGATTGCGCAAAGTATATCTTGAAG | expression of MyD88 mutant Q114A |  |
|  |  | Q114A_R | CTTCAAGATATACTTTGCGCAATCCTCCTCAATG |  |  |
| MyD88 K115A | K115A | K115A_F | GAGGAGGATTGCCAAGCATATATCTTGAAGCAG | expression of MyD88 mutant K115A |  |
|  |  | K115A_R | CTGCTTCAAGATATATGCTTGGCAATCCTCCTC |  |  |
| MyD88 Y116A | Y116A | Y116A_F | GAGGATTGCCAAAAGGCCATCTTGAAGCAGCAG | expression of MyD88 mutant Y116A |  |
|  |  | Y116A_R | CTGCTGCTTCAAGATGGCCTTTTGGCAATCCTC |  |  |
| MyD88 I117A | I117A | I117A_F | GAGGATTGCCAAAAGTATGCATTGAAGCAGCAG | expression of MyD88 mutant I117A |  |
|  |  | I117A_R | CTGCTGCTTCAATGCATACTTTTGGCAATCCTC |  |  |
| MyD88 L118A | L118A | L118A_F | GATTGCCAAAAGTATATCGCAAAGCAGCAGCAGG | expression of MyD88 mutant L118A |  |
|  |  | L118A_R | CCTGCTGCTGCTTTGCGATATACTTTTGGCAATC |  |  |
| MyD88 Ala2 A116Y | Ala2 A116Y | Ala2A116Y_F | AGGATTGCGCCGCGTATGCAGCGAAGCAGCA | expression of MyD88 mutant Ala2 with reconstituted Y116 |  |
|  |  | Ala2A116Y_R | TGCTGCTTCGCTGCATACGCGGCGCAATCCT |  |  |
| MyD88 R32A | R32A | R32A_F | CATGCGAGTGCGGCGCGCGCTGTCTCTGTTCTTG | expression of MyD88 mutant R32A |  |
|  |  | R32A_R | CAAGAACAGAGACAGCGCGCGCCGCACTCGCATG |  |  |
| MyD88 E104A | E104A | E104A_F | GACGACGTGCTGCTGGCACTGGGACCCAGCATTG | expression of MyD88 mutant E104A |  |
|  |  | E104A_R | CAATGCTGGGTCCCAGTGCCAGCAGCACGTCGTC |  |  |
| MyD88 R32AY116A | R32Y116AA | * Construct prepared by stepwise mutagenesis using primers described above. | | expression of MyD88 mutant R32A Y116A |  |
|  |  |  |  |  |  |
| MyD88 E104AY116A | E104Y116AA |  |  | expression of MyD88 mutant E104A Y116A |  |
|  |  |  |  |  |  |
| MyD88 R32AE104AY116A | R32E104Y116AAA |  |  | expression of MyD88 mutant R32A E104A Y116A |  |
|  |  |  |  |  |  |
